# Cilostazol induces cellular senescence and confers resistance to etoposide-induced apoptosis in articular chondrocytes

**DOI:** 10.3892/ijmm.2012.892

**Published:** 2012-01-23

**Authors:** KANG MI KIM, JONG MIN KIM, YOUNG HYUN YOO, JEUNG IL KIM, YOUNG CHUL PARK

**Affiliations:** 1 Department of Microbiology and Immunology, Pusan National University School of Medicine, Yangsan, Gyeongnam 626-870; 2 Department of Anatomy and Cell Biology, Dong-A University School of Medicine, Busan 602-714; 3 Department of Orthopedic Surgery, Pusan National University Hospital, Busan 602-739, Republic of Korea

**Keywords:** cilostazol, chondrocytes, dedifferentiation, senescence, apoptosis

## Abstract

We recently reported that cilostazol protects chondrocytes against stress-induced apoptosis and prevents cartilage destruction in an osteoarthritis (OA) model. In the present study, we elucidate the mechanism underlying the protective effect induced by cilostazol against stress-induced apoptosis in chondrocytes. Cilostazol significantly reduced the expression of type II collagen and stimulated the accumulation of β-catenin in primary rat articular chondrocytes. Moreover, cilostazol-induced chondrocytes showed induction of senescent phenotypes, such as changes in cell morphology, decrease in cell proliferation and increase in specific senescence-associated β-galactosidase (SA-β-gal) staining. Moreover, dedifferentiated chondrocytes obtained by serial subculture showed cellular senescence that increased with passage number. In addition, the percentage of terminal dUTP nick end-labeling (TUNEL)-positive cells was higher when chondrocytes were treated with cilostazol and the apoptosis inducer etoposide than when the cells were treated with etoposide alone. Our findings suggest that cilostazol induces dedifferentiation and senescence in rat articular chondrocytes and renders them resistant to etoposide-induced apoptosis.

## Introduction

The stability of articular cartilage depends on the biosynthetic activities of chondrocytes, which are formed by the differentiation of mesenchymal cells during embryonic development (1,2). Chondrocytes are the only cells found in articular cartilage. They synthesize appropriate extracellular matrix (ECM) molecules to maintain cartilage homeostasis (3,4). Cartilage ECM molecules such as type II collagen and sulfated proteoglycan play a crucial role in regulating chondrocyte functions by facilitating cell-matrix interactions (5). Loss of chondrocyte activity is associated with the degradation of articular cartilage in the cases of cartilage diseases such as osteoarthritis and rheumatoid arthritis, eventually leading to joint destruction (6–8).

Senescent cells remain metabolically active and show altered expression of regulatory proteins that regulate survival and proliferation. Cellular senescence is classified into 2 types. Intrinsic replicative senescence is associated with the changes in DNA structure and function, including progressive telomere shortening (9). In contrast, extrinsic telomere-independent senescence results from diverse stimuli, including ultraviolet radiation, oxidative stress, oncogene activation, and proinflammatory cytokines (10–12). These stimuli cause extrinsic stress-induced senescence in articular chondrocytes. Chondrocyte senescence plays an important role in aging and articular cartilage degeneration (13). Senescent chondrocytes accumulate with age in articular cartilage, and a correlation between increasing age and incidence of osteoarthritis has been noted (9,14).

Cilostazol is known to increase the intracellular level of cyclic AMP by blocking its hydrolysis by phosphodiesterase type III (15). Cilostazol functions as a platelet aggregation inhibitor (15) and vasodilator (16) and is mainly used for treating patients with peripheral arterial disease (17) and intermittent claudication (18). Our recent study showed that cilostazol protects rat chondrocytes against nitric oxide (NO)-induced apoptosis and prevents cartilage destruction in a rat model of osteoarthritis (OA) (19). The apoptotic effect of cilostazol in synovial cells from rheumatoid arthritis patients has also been reported (20). However, the role of cilostazol in the development, maintenance, and degeneration of articular cartilage is not known.

We found that cilostazol reduces the levels of phenotypic markers of differentiation, such as type II collagen and induces cellular senescence in primary rat articular chondrocytes. We also showed that cilostazol-induced senescent chondrocytes are resistant to etoposide-induced apoptosis.

## Materials and methods

### Reagents

Cilostazol (OPC-13013) was generously donated by Otsuka Pharmaceutical (Tokushima, Japan). Protease inhibitor cocktail, trypan blue (0.4%), 5-bromo-4-chloro-3-indolyl β-D-galactoside (X-gal), glutaraldehyde, formaldehyde, potassium ferrocyanide, potassium ferricyanide, and etoposide were purchased from Sigma-Aldrich Chemical Co. (St. Louis, MO, USA). Fetal bovine serum (FBS), Dulbecco’s modified Eagle’s medium (DMEM) and other culture reagents were purchased from Hyclon (Logan, UT, USA). Anti-poly(ADP-ribose) polymerase (PARP), Bax, type II collagen, β-catenin, and β-actin antibodies were obtained from Santa Cruz Biotechnology (Santa Cruz, CA, USA). The secondary horseradish peroxidase (HRP)-conjugated antibody and the enhanced chemiluminescence (ECL) Western blotting kit were obtained from Amersham Pharmacia Biotech (Piscataway, NJ, USA).

### Cell culture of articular chondrocytes

Articular chondroctyes for primary culture were isolated from slices of knee joint cartilage of 5-week-old female Sprague-Dawley rats (Samtako BioKorea, Osan, Korea). Chondrocytes were isolated by enzymatic digestion for 1 h with 0.2% type II collagenase in DMEM. The chondrocytes were briefly centrifuged, and the cells were resuspended in DMEM supplemented with 10% heat-inactivated FBS and antibiotics (50 U/ml penicillin, 50 μg/ml streptomycin) at 37°C with 5% CO_2_ in air atmosphere. Cells were plated on culture dishes at a density of 5×10^4^ cells/cm^2^. The medium was replaced every 2 days, and cells reached confluence at ~4–5 days after culture; this was designated as passage 0 (P0). The P0 cells were serially subcultured up to P6 by plating cells at a density of 5×10^4^ cells/cm^2^.

### Evaluation of cell viability

Cell viability was determined using the trypan blue exclusion assay. Chondrocytes were plated at 1×10^5^ cells per 6-well plates and incubated for 24 h. Cells were cultured for different times in the presence or absence of various concentrations of cilostazol in fresh DMEM medium. After incubation, the cells were washed with phosphate-buffered saline (PBS), and viable cells were scored by the trypan blue dye exclusion assay by a hemocytometer.

### TUNEL assay for the detection of apoptotic cells

Cells were washed with 1% PBS/BSA and fixed in 4% paraformaldehyde for 15 min. Next, they were washed with PBS/BSA and permeabilized in 0.1% Triton-X 100 for 5 min on ice. Fluorescein isothiocyanate (FITC)-conjugated dUTP was used for the terminal deoxynucleotidyl transferase-mediated dUTP nick end-labeling (TUNEL) assay that was performed using the Apoptosis Detection System kit (Roche Molecular Biochemicals, Mannheim, Germany) according to the manufacturer’s instructions.

### Senescence-associated β-galactosidase (SA-β-gal) staining assay

SA-β-gal staining assay was performed at pH 6.0 as described by Dimri *et al*, with a modification (21). Cells were washed in PBS, fixed for 5 min (room temperature) in 0.2% glutaraldehyde/2% formaldehyde, washed in PBS, and incubated with SA-β-gal stain solution (1 mg/ml X-Gal) 40 mM citrate/phosphate buffer (pH 6.0), 5 mM potassium ferrocyanide, 5 mM potassium ferricyanide, 150 mM NaCl, and 2 mM MgCl_2_) in a chamber maintained at 37°C for 12 h. The degree of senescence-associated cells was calculated as a percentage of the total number of cells.

### RNA isolation and RT-PCR

Chondrocytes (1×10^6^ cells/cm^2^) were grown in 60-mm culture dishes, and incubated for 24 h in fresh medium with or without cilostazol. Next, total RNA was isolated using TRIzol reagent and reverse transcription was performed using superscript reverse transcriptase (Invitrogen, Carlsbad, CA, USA) according to the manufacturer’s instructions. Total RNA (2 μg) was used to prepare cDNA. The following primers were used in our study: type I collagen, forward, 5′-GACCCAAAGGTTCTCGTGGT-3′ and reverse, 5′-CTTTCTCCTCTCTGACCGGG-3′; type II collagen, forward 5′-GGTAAGTGGGGCAAGACCAT-3′ and reverse 5′-TTTTGCAGTCTGCCCAGTTC-3′; glyceraldehyde 3-phosphate dehydrogenase (GAPDH), forward, 5′-TGAAGGTCGGAGTCAACGGATTTGGT-3′ and reverse, 5′-CATGTGGGCCATGAGGTCCACCAC-3′. PCR reactions in 25 μl reaction volumes were performed as follows: 95°C for 5 min, 30 cycles of 94°C for 30 sec, either 52°C (type I collagen) or 56°C (type II collagen) for 30 sec, 72°C for 1 min, and 72°C for 5 min.

### Western blot analysis

Equivalent amounts (20 μg) of total proteins were loaded onto 12% sodium dodecyl sulphate (SDS)-polyacrylamide gels for electrophoresis. The proteins were then transferred onto a nitrocellulose membrane by using an electroblotting apparatus (Bio-Rad, Richmond, CA), and the membranes were incubated with each primary antibody. The blots were washed with TBS-T and incubated with an HRP-conjugated secondary anti-rabbit antibody. The membranes were developed using the ECL reaction system and visualized using the LAS-3000 Luminescent Image Analyzer (FujiFilm, Japan). Image Gauge Ver. 3.0 software was used to calculate the changes in protein expression, and β-actin was used as an internal control to ensure equal protein sample loading.

### Immunofluorescence staining

Chondrocytes were cultured on collagen-coated 4-well glass chamber slides for 48 h in DMEM containing 10% FBS. Cells were fixed in 4% paraformaldehyde/PBS, followed by permeabilization in 1% Triton X-100/PBS. Appropriate primary antibodies were added to the cells for 30 min at 37°C. For secondary labeling, cells were incubated with FITC-conjugated secondary antibody (Invitrogen) for 30 min at 37°C. Nuclei were counterstained using propidium iodide (PI). Fluorescent images were observed and analyzed using a laser-scanning confocal microscope.

### Statistics or reproducibility

Each experiment was repeated at least 3 times. Data were expressed as the means ± SE from each independent experiment. The data for the experimental and control groups were tested for statistical significance by one-tailed Student’s t test, with P<0.05 accepted as the level of significance.

## Results

### Cilostazol induces dedifferentiation in primary rat articular chondrocytes

**Figure 1:**
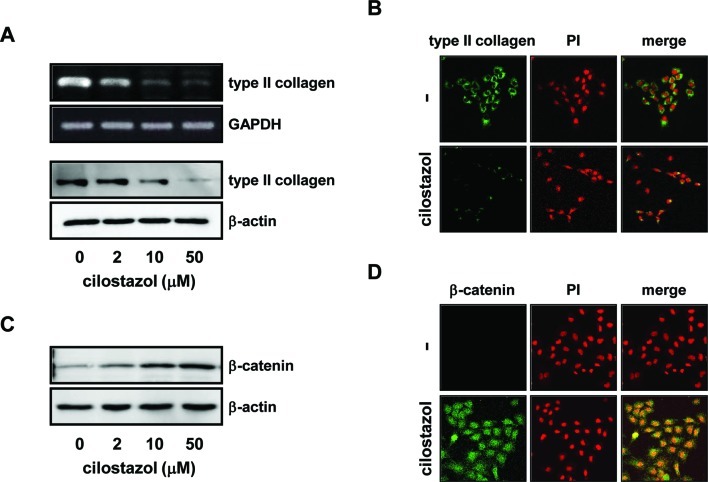
Effect of cilostazol on the expression of phenotypic markers in chondrocytes. Rat articular chondrocytes were treated with different concentrations of cilostazol for 24 h. (A) Levels of type I and II collagen were analyzed by RT-PCR and western blot analysis. GAPDH was used as an internal control. β-actin was used as an internal control to ensure equal protein loading. (B) The cells were fixed and reacted with type II collagen antibody for immunofluorescence staining by confocal microscopy. The right panels show superimposed β-catenin antibody and propidium iodide (PI) immunoreactivity (orange). (C) Western blot analysis with β-catenin antibody. (D) Immunofluorescence staining with β-catenin antibody.

To determine the role of cilostazol in the maintenance of chondrocyte phenotypes, we first investigated the effect of cilostazol on the expression of type II collagen in chondrocytes. Primary rat articular chondrocytes were isolated, maintained, and treated with various concentrations (2, 10, and 50 μM) of cilostazol for 24 h. As shown in Fig. 1A and B, cilostazol significantly reduced the expression of type II collagen, which plays a crucial role in regulating chondrocyte functions by facilitating cell-matrix interactions. In addition, cilostazol stimulated the accumulation of β-catenin, a phenotypic marker for the differentiation and dedifferentiation of chondrocytes (Fig. 1C and D). These results suggested that cilostazol causes cellular dedifferentiation, i.e., loss of differentiated chondrocyte phenotypes, in primary articular chondrocytes.

### Cilostazol-induced dedifferentiation enhances cellular senescence in chondrocytes

**Figure 2:**
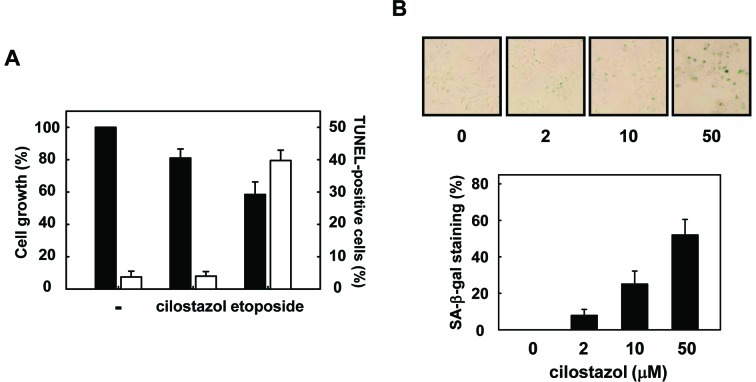
Effect of cilostazol on cellular senescence in articular chondrocytes. (A) Rat articular chondrocytes were treated with 50 μM cilostazol or 200 μg/ml etoposide for 24 h (■, total cells; □, apoptotic cells). The cells were assessed for apoptosis by TUNEL assay. TUNEL-positive cells were counted under a confocal microscope; 250–300 cells were counted for each condition. Three independent experiments were performed, and values are represented as means ± SD from the 3 experiments. (B) Rat chondrocytes were treated with different concentrations of cilostazol for 48 h and stained for SA-β-gal activity; representative images were obtained at ×20 magnification. Percentages of SA-β-gal-positive cells were determined from the numbers of blue cells per 200 cells in a randomly selected area.

Next, we assessed whether the cilostazol-induced dedifferentiation is associated with cellular senescence in primary chondrocytes. Cilostazol decreased cell proliferation without affecting cell viability (Fig. 2A). P0 cells were treated with various concentrations (2, 10, and 50 μM) of cilostazol for 48 h. Then, the induction of cellular senescence was confirmed by an increase in SA-β-gal-positive cells in cilostazol-treated chondrocytes compared to those in the control. Interestingly, cilostazol-treated chondrocytes showed significant increase in specific SA-β-gal staining (Fig. 2B).

### Dedifferentiated chondrocytes are sensitive to cellular senescence by cilostazol

**Figure 3:**
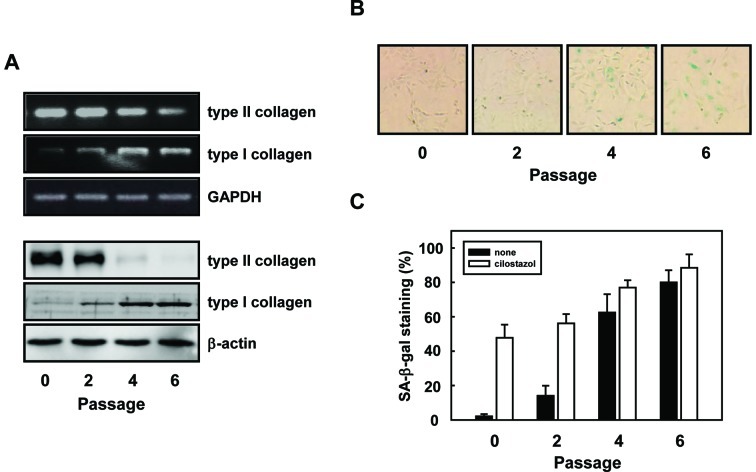
Effect of cilostazol on subculture-induced dedifferentiation of chondrocytes. Primary articular chondrocytes were serially subcultured up to 6 passages. (A) Whole cell extracts were analyzed by RT-PCR, and western blot analysis was performed using appropriate primers and antibodies, as indicated. GAPDH and β-actin were used as internal controls for the RT-PCR and western blot analysis, respectively. (B) Chondrocyte senescence was confirmed using a SA-β-gal activity assay. (C) P0, P2, P4, and P6 cells were treated with 50 μM cilostazol for 48 h, and cellular senescence was assessed by SA-β-gal staining. SA-β-gal-positive cells were counted under a microscope; 200 cells were counted in a randomly selected area.

To investigate whether cellular senescence is associated with the differentiation or dedifferentiation status of chondrocytes, we determined the effect of cilostazol on cellular senescence during subculture-induced dedifferentiation of chondrocytes. Primary chondrocytes were serially subcultured to induce cellular dedifferentiation. As shown in Fig. 3A, the expression of type I and type II collagens was significantly increased and decreased in P4 and P6 cells, respectively. The expression of β-catenin showed a similar pattern as type II collagen (data not shown). Importantly, serial subculture of chondrocytes also induced changes in senescent phenotypes, such as changes in cell morphology, decreases in cell proliferation, and increases in specific SA-β-gal staining (Fig. 3B). This suggests that chondrocyte senescence also increased during subculture-induced dedifferentiation.

Next, we observed cellular senescence in chondrocytes at different passages (P0, P2, P4, and P6) by subculture. Dedifferentiated chondrocytes showed significant cellular senescence that increased with passage number. This suggested that dedifferentiated chondrocytes slowly and spontaneously progressed to cellular senescence. Cilostazol significantly increased the SA-β-gal staining of P2 cells, and slightly increased the SA-β-gal staining of P4 and P6 cells (Fig. 3C). Cilostazol had a weak effect on senescence in dedifferentiated chondrocytes at high passage number. This is because with increasing passage number increases, senescence in cells increases. Taken together, these results suggest that senescence in chondrocytes is associated with the dedifferentiation status, which indicates the loss of differentiated chondrocyte phenotypes.

### Cilostazol renders chondrocytes resistant to etoposide-induced apoptosis via cellular senescence

**Figure 4:**
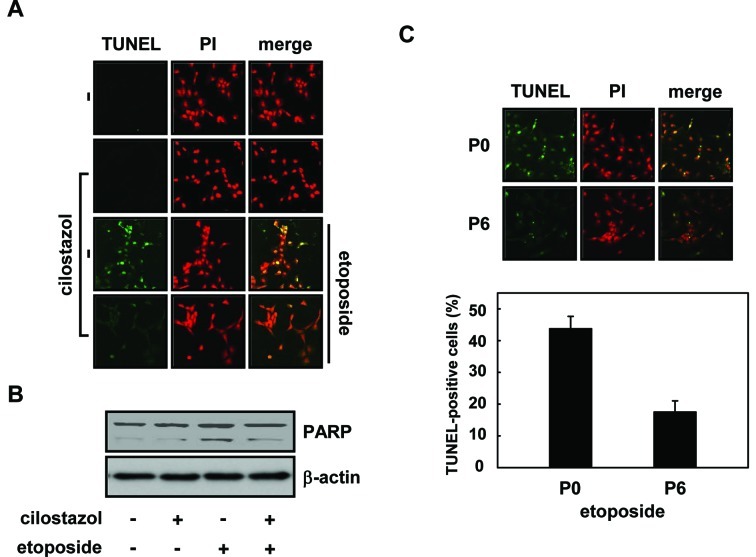
Effect of cilostazol on the etoposide-induced apoptosis in chondrocytes. Rat chondrocytes were pretreated with 50 μM cilostazol for 48 h, and then incubated with 200 μg/ml etoposide for another 24 h. (A) The cells were assessed for apoptosis by TUNEL assay. The merged images, resulting from the overlap of TUNEL-positive (green) and PI labeled (red) areas, were observed in the cells. (B) Cell lysates were analyzed by western blot analysis to detect the level of PARP cleavage (C) P0 and P6 cells were treated with 200 μg/ml etoposide for 24 h, and apoptosis was assessed by TUNEL assay. TUNEL-positive cells were counted under a confocal microscope; 250–300 cells were counted for each condition.

TUNEL assay was used to determine whether cilostazol-induced dedifferentiation and senescence changed the susceptibility of articular chondrocytes against apoptosis mediated by an apoptosis-inducing drug. Cells were pretreated with cilostazol for 48 h, and then stimulated with 200 μg/ml etoposide for an additional 24 h. The percentage of TUNEL-positive cells was markedly reduced when chondrocytes were treated with cilostazol and etoposide compared to when they were treated with etoposide alone (Fig. 4A). Etoposide is known to induce poly(ADP-ribose) polymerase (PARP) cleavage, and etoposide-induced PARP cleavage was reduced in cilostazol-treated cells (Fig. 4B). To show that senescent cells are more resistant than young cells, we used P0 and senescent P6 cells. As shown in Fig. 4C, when cells were stimulated with etoposide for 24 h, ~45 and 19% of P0 and P6 cells were TUNEL-positive cells, respectively. These results suggest that cilostazol renders chondrocytes resistant to the apoptosis inducer by inducing cellular senescence.

## Discussion

Chondrocyte apoptosis plays an important role in the degeneration and degradation of articular cartilage in the cases of osteoarthritis (OA) (22,23) and rheumatoid arthritis (7,24). We previously showed that cilostazol protects rat chondrocytes against NO-induced apoptosis *in vitro* and prevents cartilage destruction in mono-iodoacetate (MIA)-induced OA in a rat model expressing inducible NO synthase (iNOS) (19). Recent evidence suggests that cilostazol inhibits apoptosis under various conditions (25–27). This raises a possibility that cilostazol can be used for treating diseases associated with apoptotic cell death. Therefore, the precise mechanism underlying the cilostazol mediated maintenance and induction of cell death in chondrocytes of articular cartilage needs to be elucidated.

In the present study, we showed that cilostazol accelerates cellular dedifferentiation as well as cellular senescence in primary rat articular chondrocytes. This finding is supported by the following observations. As shown in Fig. 1, cilostazol significantly reduced the expression of type II collagen and stimulated the accumulation of β-catenin, which are typical phenotypic markers of chondrocyte differentiation and dedifferentiation (5,28–30). This suggests that cilostazol induces cellular dedifferentiation in primary articular chondrocytes. To confirm this finding, we also analyzed the changes in the levels of phenotypic markers during subculture-induced dedifferentiation of chondrocytes. The expression of type II collagen was completely abolished and that of type I collagen was significantly increased in P4 and P6 cells. There was no increase in the level of type I collagen in cilostazol-induced dedifferentiated chondrocytes. However, serial subculture of primary chondrocytes resulted in a decrease of cell proliferation causing changes in cell morphology in a passage-dependent manner. Therefore, we thought that the dedifferentiated state of chondrocytes could be related to cellular senescence in cilostazol-treated or subculture-induced chondrocytes.

Cellular senescence refers to a state when somatic cells enter a state of permanent growth arrest, resulting in progressive functional decline and eventual death. Senescent cells are characterized by an enlarged, flattened morphology and SA-β-gal expression (21,31). Senescent cells remain metabolically active and are resistant to apoptosis induced by exposure to genotoxic stress for a long period (32). Chondrocyte senescence causes a decline in chondrocyte numbers due to apoptotic cell death and is important in the development and progression of OA (33,34). In fact, senescent chondrocytes accumulate with age or in the cases of OA in the articular cartilage (9,13,14). Therefore, we investigated cellular senescence by conducting SA-β-gal staining assay in cilostazol-treated chondrocytes. As shown in Fig. 2, there was a significant increase in SA-β-gal staining in chondrocytes treated with cilostazol. In addition, etoposide-induced apoptosis was also reduced in cilostazol-treated or subculture-induced chondrocytes.

In conclusion, our results suggest that cilostazol induces cellular dedifferentiation and senescence in rat articular chondrocytes and render them resistant to apoptosis induced by genotoxic stress. Further studies are needed to clarify the *in vivo* effects of cilostazol on the dedifferentiation and senescence of chondrocytes in the articular cartilage of cilostazol-treated rats.

## Abbreviations

SA-β-gal: senescence-associated β-galactosidase
ECM: extracellular matrix
NO: nitric oxide
OA: osteoarthritis
TUNEL: terminal deoxynucleotidyl transferase-mediated dUTP nick end-labeling
PARP: poly(ADP-ribose) polymerase
PI: propidium iodide
ECL: enhanced chemiluminescence
